# Mapping the “housing with care” concept with stakeholders: insights from a UK case study

**DOI:** 10.1108/JICA-12-2017-0046

**Published:** 2018-10-15

**Authors:** Annie Wild, David Clelland, Sandy Whitelaw, Sandy Fraser, David Clark

**Affiliations:** 1 School of Interdisciplinary Studies, College of Social Sciences, University of Glasgow, Dumfries, UK; 2 School of Health, Wellbeing and Social Care, The Open University, Milton Keynes, UK

**Keywords:** Planning, Integrated care, Housing with care, Care campus

## Abstract

**Purpose:**

The purpose of this paper is to present the findings of an early stage, exploratory case study of a proposed housing with care initiative (the Crichton Care Campus (CCC)). This sought the perspectives of a range of key stakeholders on the proposed model and how it might be best realised. The analyses of these findings show their relevance to debates on integrated housing with care, and reflect on the methodology used and its potential relevance to similar projects.

**Design/methodology/approach:**

The study used a transactive planning approach, where grounded views are sought from a variety of stakeholders. A purposive sample identified informants from relevant health, social care and housing organisations and nine semi-structured interviews were conducted. These were transcribed and data analysis was undertaken on an “interactive” basis, relating care theory to empirical expressions.

**Findings:**

The authors identify two contrasting orientations – inclusive “community-oriented” and professional “service-oriented”. This distinction provides the basis for a rudimentary conceptual map which can continue to be used in the planning process. Two significant variables within the conceptual map were the extent to which CCC should be intergenerational and as such, the degree to which care should come from formalised and self-care/informal sources. The potential to achieve an integrated approach was high with stakeholders across all sectors fully supporting the CCC concept and agreeing on the need for it to have a mixed tenure basis and include a range of non-care amenities.

**Originality/value:**

This paper offers originality in two respects. Methodologically, it describes an attempt to undertake early stage care planning using a needs led transactive methodology. In more practical terms, it also offers an innovative environment for considering any approach to care planning that actively seeks integration – based on an acknowledgement of complexity, a variety of perspectives and possible conflicts. The authors propose that the concepts of “community-orientation” and “service-orientation” are used as a helpful basis for planning negotiations, making implicit divergences explicit and thus better delineated.

## Introduction

Whilst interest in health and social service integration (Hutchinson, 2015) and specifically integrated care (Glasby and Dickinson, 2006) has developed at pace, there has been relatively little concern for housing in this context (Glasby *et al.*, 2014). This is starting to be redressed with the “rediscovery” of housing as a social policy priority (Bramley, 2007) and the recognition of profound associations between housing and health (Prochorskaite and Maliene, 2013). More specifically, recent years have also seen growing interest in “housing with care” (Croucher *et al.*, 2006) that seeks to identify optimal housing and care arrangements. Nevertheless, perceptions exist that the quality of stakeholder integration across health, social care and housing domains remains poor – existing in “separate worlds” (Glasby *et al.*, 2014). This results in restricted planning processes and a failure to explore the variety of forms that housing with care might take (Blood, 2013). The need for a more inclusive approach to planning is therefore suggested (Whittemore, 2015).

In this context, this paper reports on a scoping study that brought together the perspectives of stakeholders from health, care and housing sectors. The proposed development is the Crichton Care Campus (CCC), which would be built within the grounds of the Crichton Campus in Dumfries, Scotland. This vision was first articulated in 2015 as “a community of people accommodated on environmentally sensitive and sustainable lines […] taking advantage of and contributing to the existing and growing amenities that exist there” (Clark, 2015, p. 1). Given that Dumfries and Galloway (D&G) is predominantly rural with high proportions of older people, the need for extensive, high quality and innovative housing with care facilities was seen as particularly acute.

The paper starts by establishing the relevant policy contexts for “healthy ageing” and “ageing in place”. It then considers insights from a series of semi-structured interviews, through the prism of a series of theoretical variables considered significant in shaping the eventual nature of any housing with care model. In the context of the extent to which integration is possible, it then identifies areas of consensus as well as differences in the potential vision of the concept. These differences are articulated in relation to two key variables – a broad based and inclusive community orientation or a more focussed service-led approach. Also having local significance, the paper proposes that the planning for any housing with care development would benefit from this integrated approach that sought to explore and understand stakeholders’ perspectives.

## Policy context

A policy crisis in publicly provided health and social services is generally accepted (Oxford Martin Commission, 2013) with population ageing, demographic and epidemiologic transitions fuelling concerns over the long-term viability of labour and capital intensive services (Silcock and Sinclair, 2012). As such, various innovations have been suggested, including the need for whole community approaches (Woodhouse, 2015), higher levels of health and social care integration (Burgess, 2016) and personalised care (Pearson *et al.*, 2014). The significance of the design of communal living environments has also increasingly been recognised within a growing policy confluence of achieving integrated health, social and housing needs (Blood, 2013). This has been specifically expressed in notions of “healthy ageing” (Steptoe *et al.*, 2014), “ageing in place” (Wiles *et al.*, 2011) and the overarching term, “housing with care” (Croucher *et al.*, 2006).

This ground seeks to identify optimal housing and care arrangements for older people and has been reflected in a range of models and terms – retirement villages, extra care and sheltered housing, and of particular interest to this paper, continuing care retirement communities that include university-based retirement communities (Howe *et al.*, 2013). Such developments tend to provide appropriate housing in association with a range of integrated health, social, personal and leisure facilities (Pannell *et al.*, 2011). Those based alongside universities seek to exploit the potential of such institutions, particularly intergenerational interactions (Demos, 2014). Perhaps, the most prominent example of this format is Schlegel Villages in Canada (Schlegel Villages, 2017).

Whilst numerous studies show that individuals living in well-designed and resourced housing with care settings tend to experience relatively higher levels of health and well-being (Evans and Vallelly, 2007; Bäumker *et al.*, 2010; Lea, 2014), others suggest that poorly planned examples can result in “grey ghettos”, isolated residents and poor health and social outcomes (Ayalon and Green, 2012). Additionally, it is recognised that such facilities are not always planned in: rational (Healey, 1992) and integrated (Glasby *et al.*, 2014) ways; or on a needs-sensitive (Kovacic *et al.*, 2015) and sustainable (Lehning *et al.*, 2015) basis. These recognitions formed the rationale for our methodology.

## Methodology

To ensure the earliest stages of planning were open and informed by the needs of all relevant parties, the project steering group formulated three complementary pieces of foundational work: a literature review on the nature of “housing with care”; a focus group-based “care needs assessment” exercise with older people; and exploratory research that sought to establish broad insights from a range of key stakeholders. This paper reports on this latter element. In relation to planning theory, Whittemore (2015) has suggested a series of potential stances that range from the top down authority of a “rational-comprehensive” approach to a “humanist” model that foregrounds the views of those on the ground. Due to the open and potentially complex nature of the CCC, an approach that Whittemore (2015, p. 78) termed “transactive” was chosen – “an ongoing practice of knowledge exchange (where the) transactive planner (is) open minded […] eschewing the idealised end states of rational comprehensive planners”.

The aim of this strand of work was to explore the preliminary potential parameters of the CCC concept. We did this by initially drawing on the housing-related literature (Lund, 2017) that identified these key dimensions, then to use these as a basis of an interview schedule. We drew up an intentionally broad purposive sample of stakeholders including senior staff from D&G Council and NHS D&G in the areas of housing, social work, community planning, and health and social care integration. The sample also included staff from a body that represents independent health and social care providers; a local third sector interface organisation; and a national dementia charity. Semi-structured interviews were undertaken with nine individuals.

The interviews were designed to be relatively open-ended, with stakeholders having a full view of the schedule, enabling them to pick out themes they each felt were most relevant to further defining the CCC. With a few exceptions, stakeholders expressed perspectives on all of these themes but some stood out as consistently gaining most attention. Through an iterative process of initial coding into categories, discussion, then recoding, four overarching analytical themes were generated that address the key definitional issues pertinent to the possible nature of CCC. This mapping of the “housing with care” concept draws on a methodology seen by Trochim and Kane (2005, p. 187) as being “used to produce a picture or map of the ideas or concepts […] [providing a] framework or structure that can immediately be used to guide action planning, programme development”.

## Analytical themes

### Intergenerationality

A prominent theme in the literature concerns the age range of residents and the extent of community inclusivity (Cole and Goodchild, 2000). Some have suggested that accommodating residents with a wide age range is mutually constructive for both (relatively) younger and older groups (Darton and Muncer, 2005) wherein, “an optimal psychological group equilibrium” (Baker, 2002, p. 26) is realised. A principle of inclusion – in terms of age or other cultural, ethnic or social characteristics – is therefore seen by some as instrumental in avoiding the “ghettoisation” of older people (Croucher *et al.*, 2006; Harhaj, 2014). Others, however, suggest that multi-generation settings can be problematic or cause tensions (Greenwood and Smith, 1999), where ways of life appropriate to less able older people are not attractive to younger residents (Croucher *et al.*, 2003), or where residents prefer “mono-generational” settings (Bernard *et al.*, 2004).

Our informants also took a significant interest in this theme. They were broadly supportive of an inclusive, intergenerational approach, suggesting that CCC should not be a “ghetto” or “warehouse”, populated entirely by older people. Rather it should be a mixed community that avoids social isolation and supports a mix of different types of care; for example, “I feel quite strongly it should be intergenerational”. However, there was no consensus on the degree to which the CCC should be intergenerational. Whilst a segregated model was unanimously rejected, a range of views existed about the degree to which the CCC should be focussed on care for older people or a much broader “model village” project. Some suggested replicating as many aspects of a “natural” community as possible; for example, “it should be very much a community […] not just a community of people with dementia but a community where there’s young families, old people, people with dementia, people not with dementia”. Others felt the emphasis needed to be on providing care to a more focussed group of people and too many non-specialist facilities could subvert the project’s purpose; for example, reflecting on the idea of having a nursery on campus an informant suggested, “if it was focused […] on children with particular needs […]. I think that would be consistent with the concept […] but if it was just another children’s nursery then we’ve got them across the region”.

### Types of care

A similar debate exists around the nature and levels of morbidity and disability that could be catered to within this care setting (Darton and Muncer, 2005) and again this theoretical ground was significant to our informants. Some academics believe that “housing with care” should be based on relatively open principles, including some individuals without any particular or definable morbidity or disability (Baker, 2002). Another perspective is that “housing with care” facilities should meet needs of those with significant morbidities and disabilities (Roth *et al.*, 2011) including the provision of palliative care (Tanuseputro *et al.*, 2017). The notion of “dementia-friendly communities” advocates a planning approach that is tailored to those with the condition but often through more informal types of care and a high level of community awareness (Waller *et al.*, 2017).

The provision of care of some sort is a central feature of any housing with care initiative and makes such initiatives attractive to potential residents (Sherwood *et al.*, 1997). In an ideal and uncritical context, and underpinning the acceptance of a full “continuum of care needs” (McNabney *et al.*, 2009), such care would be comprehensive and varied, spanning high and low intensity and health and wider social interventions (Pannell *et al.*, 2011). Descriptions in the literature cite a whole host of possibilities of formal care services, e.g. primary healthcare, general nursing care, assisted living support, psychological services and memory care.

Informants possessed a general view that CCC should indeed be aimed at older adults with care needs. In particular, most felt that dementia care needs should be central to CCC infrastructure planning and services couched by one informant as a “very, very, very dementia friendly” community. Many also believed that CCC should have the resources to provide for people at the end of life, potentially with options to move to different accommodation within the grounds; for example, “we should be offering people care right to the very end stages […]. I don’t think there should be a definition as to whether there’d be a cut-off point, or you need to be moved on to some other kind of care establishment […]. I think if you’re having a care campus it’ll have to encompass everything that goes along with that”. Stakeholders generally supported the need for a mix of care types and intensity of care, being able to cater to people with a range of needs; for example, “I think we need a whole spectrum of care”.

The inclusion of specific healthcare services (e.g. podiatry, optician, dentistry, hygienist and minor injuries treatment) was seen as positive, though there were some concerns that an overtly “medicalised” and “high-intensity” development might not be compatible with wider goals of providing a “homely” social setting. This was felt particularly pertinent given that the proposed location of CCC would be close to some existing NHS services. One informant attempted to square the tension between these perspectives in proposing, “almost like [formal] carers are kind of hovering in the background […] and it’s there and it’s supporting […] but it’s almost unseen”. Drawing on the above inter-generation ethic, some believed that there should be options for families being accommodated on the campus to facilitate unpaid care; for example, “it won’t just be the person with dementia that would be moving in […] it would […] be husband, wife or perhaps a son or a daughter”. The appeal of this arrangement being that younger people could informally provide low-intensity care. Some interviewees cited examples such as those in the Netherlands where housing and care for older people is combined with accommodation for students and mechanisms such as volunteering and time-banking.

### Range of housing types

Beyond these interests in the generational make-up and the nature of the delivered care, there is a perception that the housing element of care has tended to be neglected (Demos, 2014). A central aspect of this theme is the nature of residents’ tenure. The basis of this can often be a combination of private sector and housing association (Croucher *et al.*, 2006) so that residents can be owners, tenants and leaseholders with the possibility of “mixed” forms of buying and renting tenure (Pannell *et al.*, 2011). Significantly, tenure types are seen as a helping determine the economic and social mix of residents in housing with care establishments (Pannell *et al.*, 2011).

All stakeholders strongly supported the idea of a mix of accommodation types – for one, “about as wide as it could be possibly be within the constraints of the locality” – and that accommodation should be available for those across the assets and income spectrum. There was a wide belief that home ownership should be an option given local shortages of appropriate accommodation for older people who are able to buy and a perception that current homeowners found it difficult to access housing with care. Informants noted that a more communal option should also be possible, with some advocating the notion of “co-housing”: “some people don’t want to be on their own […] don’t want to have their own house […]. I think that’s quite important that that’s an option that’s there too”.

More generally, informants acknowledged the growing demand for housing for older people and perceived shortages in D&G. This included the need for suitably adapted housing with additional support for older people and people with disabilities as well as facilities forming a “half-way house” between older people living in their own home and permanent residential care. The CCC was seen as an opportunity to pursue innovative approaches to meeting these needs and enabling residents to maintain their independence for as long as possible. The nature of housing provided was seen as a central factor; for example, “if you had various accommodation types […] some of them being adapted so that they could support people with more complex needs but who were still able to live reasonably well independently in their own home”.

### Other facilities

Finally, the literature highlights the potential for the inclusion of a wider set of social, wellness and physical activity programmes and more generic services (Croucher *et al.*, 2006) and this was a prominent theme throughout the interviews. The need for such breadth is, however, tempered by a series of moderating influences. The ability to realistically fund a comprehensive range of amenities is questioned, particularly in smaller-scale facilities (Bynum *et al.*, 2011). Also, if housing with care communities is potentially comprised of residents with varied needs, it may not be necessary to provide such a full range of in-house services (Croucher *et al.*, 2006).

All stakeholders felt that there was a need for non-care amenities such as retail, catering, a library, a gym, exercise and arts classes, internet access, spiritual care and gardens. Innovative suggestions were also offered, such as a small-holding or static caravan that could enable more vulnerable residents to go “on holiday”. Non-care facilities were seen as being important to residents’ quality of life and sense of independence, and potentially attractive to carers and other non-residents, helping to integrate CCC into the wider community; for example, “things like a restaurant and all these social things that aren’t available in care homes […] because otherwise it’s actually not really solving anything […] you might as well still just be in a big care home in the middle of nowhere”.

Not everyone shared this embedded and comprehensive vision. Some felt that older people may prefer to continue using the same services; for example, “just because you come into care why should you stop going to your own hairdressers?” The provision of adequate transport links to existing facilities in Dumfries town centre was therefore seen as a priority for these individuals.

## Discussion

Independent of sector status, respondents supported the CCC concept, with any scepticism about implementation linked with the suggested site rather than the concept *per se*. There was widespread agreement on the need for innovative housing with care models to address the multiple challenges of the aging society and for the service to cater to a range of morbidities. All respondents felt that the accommodation should offer a mix of tenure types to ensure accessibility to people from a range of income groups. There was also clear convergence on what should be avoided – CCC ought not to be a “warehouse” or “grey ghetto”.

These commonalities aside, two contrasting interpretations were identified through analysis. One reflected a broad-based and inclusive community-oriented approach, with a wide range of embedded non-care services, replicating many aspects of a “natural” community. The other was a more focussed, professional service-orientation which sought an integrated and person-centred approach to care but somewhat more healthcare focussed. These two positions help delineate a series of contrasting priorities that can inform further development of the CCC model.

First, these underpin the issue of who the CCC should fundamentally be aimed at. For those who suggested a community-oriented approach, building an inclusive ethos is considered paramount and the widest range of residents would be desirable – those with and without care needs. In contrast, for the service-oriented view, those with care needs are understood as the key user group and others would either not be included in plans, or only included in circumstances where this was of direct benefit to this user group – for example, family members or those offering paid care services.

Closely related to this issue were the different conceptions of intergenerationality. For those favouring a community-oriented model, the inclusion of younger families and other non-target groups was considered an inherent feature of CCC, implying a level of intergenerationality that goes further than usually conceived in the literature. Those reflecting a service-oriented mind-set found the concept of intergenerationality attractive in theory, but were often ambivalent or unsure about the potential role of younger people within the CCC. Some even worried that including too many people from outside the core user group could “dilute” the scheme’s purpose.

The differentiation also creates contrasting visions of the nature of care. The community-orientated model would minimise the medicalised aspects, emphasising non-professional care, self-care and organisational approaches such as mutually supportive co-housing. Given broad agreement that the widest possible range of morbidities should be catered for in the CCC, this highlights a potential tension within the community-oriented model in that intense care must somehow be offered in a way that does not lead to an overly medicalised setting. In contrast, those that favoured a service-oriented approach were less concerned with the way in which formalised care might affect the social fabric of the community. Therefore, more conventional forms of paid care were just as acceptable as these more innovative models.

In the context of integration, it is worth noting that whilst there were some variations in perspective in relation to intergenerationality and “community-” or “service-” oriented positions, empirically, we found no discernible “patterning” in relation to the health, social work, community planning, housing and care sector stakeholders we accessed. As such this domain may be one where traditional sectoral tensions are less evident and the potential for genuinely integrated work high.

## Conclusion

At the onset, we suggested that there is an acceptance of a general need for housing considerations to become a much more prominent feature in the development of optimal integrated health and social care policy. We conclude that beyond this aspiration, there is now a need to actually enact this ambition and then monitor and appraise the nature and robustness of resultant processes – how we arrive at the design of housing with care being just as important as eventual outcomes. Whilst housing is becoming a more visible feature of practical care policy, there is little evidence of much process-oriented evaluative work and associated literature, particularly in an integrated context (Clark and Whitelaw, 2017).

This paper reports on work that has sought to start to address this gap – describing the initial steps of a potentially long term, complex and multi-faceted “transactive” planning process that sought views from a range of stakeholders in an integrated environment. We suggest that the primary planning variables for a potential housing with care development are intensity of service and the extent of intergenerationality, reflecting fundamentally how specialist or broad based the future residential community on the Crichton could be. Other secondary themes (tenure, housing type and range of facilities) complement these foundations. While some options may be mutually exclusive, the contrasting “community” and “service” orientations should be seen as extremes of spectrum of possible outcomes, and this is not to discount the possibility of different combinations or options in the final outcome of any transactive process. As above, the broad congruence of stakeholder perspective, independent of sector status suggests potential for a genuinely integrated approach to housing with care. However, in later phases of the planning process, the potential for more sector-specific positions to emerge is certainly conceivable.

Through this process, we have also identified some limitations of a “transactive” approach to housing with care. The relatively open and participant-led nature of the interviews was effective at allowing participants to focus on the themes they felt were most important, preventing us from prematurely imposing a framework on the discussions. It could be argued though that this encouraged somewhat utopian thinking as stakeholders were not confronted with the viewpoints of others or practicalities such as finances or the planning system. A transactive approach to planning has also been criticised for not tackling unequal power relations (Parker and Street, 2015). However, these interviews were just one early part of a wider project and focus groups with older people are one example of how the voices of potential users can also be incorporated into the process.

A new project is now underway funded from the European Union LEADER programme, which exists to support rural development projects initiated at the local level. The project seeks to test “proof of concept” for CCC and through a programme of community engagement will develop a set of options, which will be assessed and analysed for strengths, weaknesses and potential benefits for the region, with a preferred model as the key outcome.

There is now a longer term and specific need to explore how the transition from preliminary and hypothetical expressions of housing with care to actual planning and delivery will be manifest within a complex range of “wicked” policy variables, for example: variously, expressions of “need”, “choice” and the legally backed obligation in Scotland of “personalisation”; different levels of care; different models of housing; levels of available resource; all bounded by politically and potentially varied sectoral orientations in an integrated context. In this paper, we show that a transactive planning approach is suited to this complexity and offer the prism of “community-” and “service-” oriented stances as potential means of making explicit divergent planning assumptions between different stakeholders, thus provoking debate on ways forward.
